# Podocalyxin Expressed in Antigen Presenting Cells Promotes Interaction With T Cells and Alters Centrosome Translocation to the Contact Site

**DOI:** 10.3389/fimmu.2022.835527

**Published:** 2022-05-31

**Authors:** Laura Amo, Javier Díez-García, Estíbaliz Tamayo-Orbegozo, Natalia Maruri, Susana Larrucea

**Affiliations:** ^1^ Regulation of the Immune System Group, Biocruces Bizkaia Health Research Institute, Barakaldo, Spain; ^2^ Microscopy Facility, Biocruces Bizkaia Health Research Institute, Barakaldo, Spain; ^3^ Regulation of the Immune System Group, Biocruces Bizkaia Health Research Institute, Cruces University Hospital, Barakaldo, Spain

**Keywords:** podocalyxin, dendritic cells, T cells, cell interaction, centrosome polarization, antigen presenting cells, immune synapse, immune system

## Abstract

Podocalyxin (PODXL), a cell surface sialomucin expressed in diverse types of normal and malignant cells, mediates cellular adhesion to extracellular matrix and cell-to-cell interaction. A previous study reported the expression of PODXL protein on monocytes undergoing macrophage differentiation, yet the expression of this molecule in other antigen presenting cells (APCs) and its function in the immune system still remain undetermined. In this study, we report that PODXL is expressed in human monocyte-derived immature dendritic cells at both the mRNA and protein levels. Following dendritric cells maturation using pro-inflammatory stimuli, PODXL expression level decreased substantially. Furthermore, we found that PODXL expression is positively regulated by IL-4 through MEK/ERK and JAK3/STAT6 signaling pathways. Our results revealed a polarized distribution of PODXL during the interaction of APCs with CD4^+^ T cells, partially colocalizing with F-actin. Notably, PODXL overexpression in APCs promoted their interaction with CD4^+^ T cells and CD8^+^ T cells and decreased the expression of MHC-I, MHC-II, and the costimulatory molecule CD86. In addition, PODXL reduced the translocation of CD4^+^ T-cell centrosome toward the APC-contact site. These findings suggest a regulatory role for PODXL expressed by APCs in immune responses, thus representing a potential target for therapeutic blockade in infection and cancer.

## Introduction

Professional antigen presenting cells (APCs), including dendritic cells (DCs), macrophages and B cells, are immune cells with the ability to capture, process and present antigens to T cells (1). Among them, DCs are considered the most efficient APCs (1). In contrast to macrophages and B cells, DCs possess the capacity to initiate primary immune responses by activating naïve T cells, effectively linking innate and adaptive immune responses required for protective immunity against infection and cancer (2, 3). DCs also induce and maintain central and peripheral tolerance through inhibition of T cell activation, generation of regulatory T cells, and induction of T-cell anergy or deletion to limit uncontrolled immune responses (4, 5). The tolerogenic capability of DCs relies on their maturation state, the presence of anti-inflammatory molecules and the type of microbial stimuli (6). The induction of immunity or tolerance depends on the balance between activating and inhibitory processes (7). Therefore, the identification of molecules that elicit immunomodulatory effects may provide new therapeutic strategies to treat infectious and malignant diseases (7, 8).

Immature DCs patrol peripheral tissues in search of pathogens and foreign antigens (9). They express pattern recognition receptors (PRRs) such as Toll-like receptors (TLR), nucleotide oligomerization domain (NOD)-like receptors (NLRs) and C-type lectin receptors for the detection of bacterial and viral components (10, 11). The exposure to microbes or antigens or the stimulation with inflammatory cytokines induces DCs maturation, a process characterized by the reduction in the endocytic activity, production of cytokines and up-regulation of surface major histocompatibility complex (MHC), co-stimulatory surface molecules, adhesion molecules as well as chemokine receptors (12–14). During their maturation, antigen loaded DCs migrate to lymph nodes where they interact with naïve T cells and initiate the adaptive immune response (15). The recognition of antigenic peptides presented in the context of MHC molecules by T cell receptor (TCR) displayed on T cells leads to the redistribution of cell surface receptors, adhesion molecules, and signaling proteins along with the reorganization of F-actin and microtubule cytoskeleton at the contact site, forming a structure known as immune synapse (16–18). This process is accompanied by the dynamic reorientation of centrosome, the major microtubule-organizing center, in T cells to the immune synapse, allowing the directional secretion of cytokines that mediate T-cell effector functions (19, 20).

Podocalyxin (PODXL), a cell surface protein belonging to the CD34 family of sialomucins and initially detected on renal podocytes, has been observed in endothelial cells, mesothelial cells, megakaryocytes and neuronal cells, hematopoietic stem cells as well as in a variety of tumor cell types, including blasts presenting a monocytic phenotype from patients with acute myeloid leukemia and acute lymphoblastic leukemia (21–28). In hematopoietic cells, PODXL is highly expressed by primitive erythroid precursors and early embryonic hematopoietic progenitors and its expression level gradually declines during ontogeny (26). After birth, PODXL expression only remains in a subpopulation of hematopoietic progenitor cells found in the bone marrow (26). In myeloid progenitors, PODXL expression is up-regulated upon stimulation with granulocyte colony-stimulating factor (G-CSF) (29). In mature leukocyte populations, including lymphocytes, monocytes, and granulocytes, PODXL expression has only been found at mRNA level and the attempt to detect PODXL protein expression in these leukocytic populations has yielded negative results (30). A previous study reported the expression of PODXL protein on the surface of monocytes undergoing macrophage differentiation in response to macrophage colony-stimulating factor (M-CSF) (31), although its function in these cells is currently unknown. PODXL interacts with actin through the cytoskeletal organizer ezrin, allowing it to regulate cell morphology, polarity, adhesion and migration (32, 33). Both anti-adhesive and adhesive properties have been attributed to PODXL. The high negative charge of PODXL conferred by the sialylated O-glycans provides an anti-adhesive force that maintains opened the filtration slits in glomerular podocytes (22). In contrast, PODXL expressed on the surface of high endothelial venule cells supports the tethering and rolling of circulating lymphocytes *via* interaction with L-selectin (34). PODXL also contributes to neuronal development and formation of synapses in central nervous and neuromuscular systems (25).

Recently, we demonstrated that PODXL expressed in MCF-7 breast cancer cells localizes to the immune synapse formed upon contact with NK cells and acts as an immunomodulatory molecule (35). Moreover, we detected increased expression of PODXL in breast cancer cells in response to IL-4 (35), a cytokine routinely used in combination with granulocyte-macrophage colony-stimulating factor (GM-CSF) to differentiate blood derived monocytes into DCs *in vitro* (36). However, whether PODXL is expressed by DCs and its function in the immune system still remain unaddressed. In the present study, we have determined the expression of PODXL in human DCs and studied the role of this protein in APC-T cell interaction. The results revealed that PODXL is expressed in monocyte-derived immature DCs, but its level markedly diminishes upon maturation. Furthermore, PODXL promotes APC-T cell interaction and modulates CD4^+^ T-cell centrosome repositioning to the contact site. Our results point to a role for PODXL expressed by APCs in the regulation of immune responses.

## Materials and Methods

### Cell Lines and Primary Cells

Peripheral blood mononuclear cells (PBMCs) were isolated by density gradient centrifugation over Lymphoprep (Alere Technologies AS). Monocytes were isolated from PBMCs by negative selection using the Dynabeads^®^ untouched human monocytes kit (Invitrogen) according to the manufacturer´s instructions. More than 95% of the purified cells were monocytes as confirmed by flow cytometry on the basis of CD14 expression. For the generation of immature monocyte-derived DCs, monocytes were cultured in complete RPMI 1640 medium (Lonza) supplemented with 10% heat-inactivated fetal bovine serum (FBS, Hyclone), 100 U/ml penicillin, 100 U/ml streptomycin (Lonza) or in AIMV serum-free (Gibco), both containing 400 U/ml recombinant human IL-4 (rhIL-4) and 800 U/ml recombinant human GM-CSF (rhGM-CSF) (Peprotech) for 5 or 6 days at 37°C in a 5% CO_2_ atmosphere. Half of the culture medium was removed and replaced with fresh medium containing 400 U/ml rhIL-4 and 800 U/ml rhGM-CSF on day 3. For the induction of maturation, immature monocyte-derived DCs were incubated with 100 ng/ml lipopolysaccharides (LPS) from Escherichia coli 055:B5 (Sigma-Aldrich) or a maturation cocktail containing 20 ng/ml tumor necrosis alpha (TNFα, Miltenyi Biotec), 20 ng/ml IL-1β (Miltenyi Biotec), 20 ng/ml IL-6 (Miltenyi Biotec) and 1μM prostaglandin E2 (PGE2, Sigma-Aldrich) for 48 h. CD4^+^ T and CD8^+^ T cells were separated from PBMCs by negative immunomagnetic selection using Dynabeads^®^ untouched Human CD4^+^ T cells kit (Invitrogen) and Dynabeads^®^ untouched Human CD8^+^ T cells kit, respectively.

THP-1 (acute monocytic leukemia), K562 (chronic myelogenous leukemia), HL-60 (acute promyelocytic leukemia), KG1 (acute myelogenous leukemia), Raji (Burkitt lymphoma), and Jurkat (acute T cell leukemia) human cell lines were obtained from American Type Culture Collection (ATCC). Cells were cultured in RPMI 1640 (THP-1, Raji, Jurkat) or IMDM (K562, KG-1, and HL-60) media supplemented with 10% (THP-1, K562, Raji, Jurkat) or 20% (KG-1 and HL-60) heat-inactivated FBS, 100 U/ml penicillin, and 100 U/ml streptomycin under 5% CO_2_ at 37°C. For THP-1 cell culture, 50 μM 2-mercaptoethanol was added to the medium.

### Generation of Stable Transfected Cells

Raji cells were transfected with human PODXL coding sequence subcloned into pEGFP-N1 expression vector (a gift from Dr. R. Parrilla, CIB-CSIC) or with pEGFP-N1 empty vector as a negative control using Lipofectamine 2000 (Thermo Fisher Scientific) as previously described (37). Transfected cells were routinely grown in RPMI 1640 complete medium supplemented with 400 μg/ml geneticin (Sigma-Aldrich).

### Immunoblotting

Cells were washed twice with PBS and lysed in ice-cold lysis buffer containing 1% Igepal, 1% Triton X-100, 20 mM Tris HCl HCl, pH 7.4, 140 mM NaCl, 1 mM EDTA, complete protease inhibitor cocktail (Sigma-Aldrich), 1mM phenylmethylsulfonyl fluoride, 10 mM sodium fluoride, 1 mM sodium orthovanadate, and 1 mM sodium pyrophosphate for 30 min on ice. Cell debris was removed by centrifugation at 13,000 g for 15 min at 4°C and the supernatants were collected and stored at -80°C. Protein concentration of supernatants was determined using Pierce™ BCA Protein Assay Kit (ThermoScientific) and POLARstar Omega microplate reader (BMG Labtech). Equal amounts of total protein (5-50 μg/lane) were resolved on 4%-12% SDS-PAGE gel and transferred onto iBlot pre-made polyvinylidene difluoride membranes using iBlot Dry Blotting System (Life Technologies). After blocking with 1% bovine serum albumin (BSA) in Tris-buffered saline solution with 0.1% Tween 20 (TBS-T), membranes were incubated overnight at 4°C with an anti-PODXL monoclonal antibody (Santa Cruz Biotechnology, cat.n° sc-23904) at 1:200 dilution in blocking solution, followed by a secondary goat anti-mouse IgG (H+L)-HRP-conjugated antibody (Bio-Rad, cat.n° 172-1011) at 1:3,000 dilution in TBS-T buffer containing 5% non-fat dry milk for 1 h. Immunoreactive proteins were detected by using enhanced chemiluminescence SuperSignal ™ West Femto or Pierce™ ECL Western Blotting Substrate (Thermo Sientific) in a G:BOX iChemi XR (Syngene) or ChemiDoc XRS imaging system (Bio-Rad). Equal loading of protein samples was verified with anti-GADPH (Ambion) or anti-β-actin (Sigma) antibodies. Quantification of protein expression was performed using Fiji software (ImageJ, https://imagej.net/software/fiji/).

### Real-Time PCR

RNA was extracted from 5x10^5^ cells using the RNeasy Plus Micro Kit from Qiagen according to the manufacturer’s instructions. The concentration and quality of the extracted RNA was assessed using a NanoDrop spectrophotometer (ThermoScientific). The purified RNA was stored at -80°C. Human PODXL mRNA levels were estimated by a two-step qRT-PCR using a customized TaqMan Gene Expression Assay kit (Applied Biosystems) following the manufacturer’s instructions. The human housekeeping gene HuPO (Pre-Developed TaqMan Assay Reagents, Applied Biosystems) was used as the endogenous control and all qRT-PCR reactions were performed in triplicate. qRT-PCR experiments were conducted on an Applied Biosystems 7900 HT Fast Real-Time PCR System, and the Relative Quantitation (Comparative C_T_) method was used to estimate the relative changes in gene expression using the RQ Manager v.1.2 analysis software (Applied Biosystems).

### Flow Cytometry

For flow cytometric analysis of PODXL expression, cells were resuspended in labelling solution (PBS supplemented with 0.1% BSA and 0.01% NaN_3_) and incubated with 20 μg/ml human IgG for 15 min at room temperature to block nonspecific Fc interactions. Then, cells were stained with a mouse anti-human PODXL monoclonal antibody (R&D Systems, cat.n° MAB1658) for 30 min at 4°C followed by a secondary PE-conjugated anti-mouse IgG secondary antibody (R&D Systems) for 20 min at room temperature or with a biotinylated goat anti-human PODXL antibody (R&D Systems, cat.n° BAF1658) for 30 min at 4°C followed by PE-conjugated streptavidin (Biolegend) for 20 min at room temperature, as indicated in figure captions. Isotype-matched control antibodies were used to evaluate nonspecific binding. Dead cells were stained with 7-amino-actinomycin D (7-AAD staining, BD Pharmingen). For some experiments, FITC-CD209 (BD Biosciences, cat.n° 551264) and CD14-APCH7 (BD Biosciences, cat.n° 641394) monoclonal antibodies were used to discriminate monocytes and DCs. Other monoclonal antibodies used included CD54-APC (cat.n° 353111), CD80-APC (cat.n° 305219), CD86-APC (cat.n° 305411), APC-HLA-A,B,C (cat.n° 311409), APC-HLA-DR,DP,DQ (cat.n° 361713) and CD40-PE (cat.n° 334307) from Biolegend, CD14-PE (cat.n° 555398), CD11b-APC (cat.n° 333143) and CD11c-APC (cat.n° 333144) from BD Biosciences, and CD36-FITC (cat.n° 36F2-100T) from Immunostep. Cells were then fixed with 1% formaldehyde, washed and resuspended in 700 μl PBS. Finally, stained cells were acquired on a Cytomics FC500 (Beckman Coulter) or a MACSQuant Analyzer 10 (Miltenyi). A minimum of 5.000 events per sample were acquired. Data analyses were performed with CXP (Beckman Coulter) or MACSQuantify (Miltenyi) analysis software. Dead cells were discriminated based upon 7-AAD. Data are expressed as median fluorescence intensity (MFI) corrected for nonspecific staining using fluorescence minus one and isotype controls.

### Conjugate Assay

Raji cells overexpressing PODXL or Raji control cells were incubated in the presence or absence of 2 μg/ml of the superantigen staphylococcal enterotoxin A from Staphylococcus aureus (SEA, Sigma-Aldrich) for 20 min at 37°C and resuspended at 1.5 x10^6^ cells/ml in complete RPMI 1640 medium. SEA was used to crosslink MHC class II (MHC-II) expressed on APCs to TCR expressed on T cells. Isolated CD4^+^ T cells or Jurkat T cells were labelled with CellTracker™ Orange CMTMR Dye (Invitrogen) at a final concentration of 20 μM or 5 μM, respectively, following manufacturer´s instructions, and resuspended at 1.5 x10^6^ cell/ml in complete RPMI 1640 medium. Afterwards, equal volumes (150 μl) of Raji cells and T cells were mixed, briefly centrifuged to favor cell contact, and incubated at 37°C for the indicated times. Cells were then briefly vortexed to disrupt non-specific aggregates and fixed with 1% formaldehyde for 1 h at room temperature. Twenty thousand events per sample were acquired and analyzed by flow cytometry. The percentage of Raji cell-T cell conjugation was calculated as the number of double-positive (GFP^+^CMTMR^+^) events divided by the total number of GFP^+^ cells (total Raji cells). In blocking experiments, both Raji and Jurkat cells were separately preincubated with 10 μg/mL anti-human CD18 antibody TS1/18 (Biolegend, cat.n° 302111) for 15 min at 37°C prior to mixing the cells and the antibody was present during the APC-T cell incubation.

For the simultaneous detection of both Raji-CD4^+^ T cell and Raji-CD8^+^ T cell conjugates, Raji-PODXL or Raji-Ctrl cells were mixed with PBMCs at 1:1 ratio and incubated for 30 min at 37°C. Thereafter, CD4^+^ T cells and CD8^+^ T cells were labeled with anti-CD4-APCH7 (BD Biosciences, cat.n° 641398) and anti-CD8-PE monoclonal antibodies (BD Biosciences, cat.n° 345773), respectively, in labelling solution for 20 min at 4°C. Cells were finally fixed with formaldehyde 1% in PBS for 1 h and analyzed by flow cytometry. The percentage of Raji cells-T cells conjugates was determined as the number of double-positive (GFP^+^APCH7^+^ for the detection of Raji-CD4^+^ T cell conjugates and GFP^+^PE^+^ for the detection of Raji-CD8^+^ T cell conjugates) events divided by the number of GFP^+^ cells (total Raji cells). For the conjugation assay using isolated CD4^+^ T and CD8^+^ T cells, Raji-PODXL or Raji-Ctrl cells were mixed with isolated CD4^+^ T and CD8^+^ T cells at 1:1 ratio and incubated for 30 min at 37°C. Then, CD4^+^ T cells and CD8^+^ T cells were labeled with anti-CD4-APCH7 (BD Biosciences, cat.n° 641398) and anti-CD8-APCH7 (BD Biosciences, cat.n° 641400), respectively, in labelling solution for 15 min at 4°C and proceed as described above.

### Fluorescence Microscopy

Immature DCs resuspended in Hank´s solution containing 1% BSA or immature DC conjugated with CD4^+^ T cells in complete RPMI 1640 medium were deposited onto a slide by centrifugation at 500 rpm for 3 min using a cytospin centrifuge. Next, cells were fixed with 3.7% paraformaldehyde in PBS for 15 min at room temperature and permeabilized with 0.2% saponin in PBS for 10 min at room temperature. Afterwards, Fc receptors were blocked using 20 μg/ml human IgG (Sigma-Aldrich) for 1 h at room temperature and cells were incubated with 15 μg/ml goat anti-human PODXL polyclonal antibody (R&D Systems, cat.n° AF1658) or polyclonal goat IgG antibody (R&D Systems,cat.nº AB-108-C) as control in PBS-BSA 0.1% at 4°C overnight. Finally, cells were incubated with Cy2-conjugated donkey anti-goat IgG (H+L) preadsorbed polyclonal antibody (Abcam, cat.n° ab6948) at 1:250 dilution in PBS-BSA 0.1% for 1 h at room temperature, stained with Alexa fluor 555-conjugated phalloidin (Invitrogen) at 1:60 dilution in PBS-BSA 0.1% for 20 min at 37°C for F-actin detection, followed by Hoechst 33342 for nuclei staining, and mounted in PermaFluor™ Aqueous Mounting Medium (ThermoScientific). In experiments involving immature DCs alone, cells on slides were incubated for 30 min at 37°C in a humidified atmosphere at 5% CO2 in RPMI 1640 medium containing 1% FBS prior to fixation step to allow cell migration.

For experiments involving Raji-T cell conjugates, cells were deposited onto coverslips coated with poly D-lysine (Sigma-Aldrich), fix with 3.7% paraformaldehyde in PBS for 15 min at room temperature and permeabilized with 0.5% Triton X-100 in PBS for 5 min at room temperature. Then, cells were incubated with Alexa fluor 555-conjugated phalloidin and Hoechst 33342 as described above.

For centrosome detection in Raji-T cell conjugates, cells on poly D-lysine coated coverslips were fixed with methanol at -20°C for 10 min and permeabilized with 0.1% Triton X-100 in PBS containing 0.1% FBS for 30 min at room temperature. Afterwards, cells were incubated with anti-γ-tubulin monoclonal antibody (Biolegend, cat.n° 629201) at 1:500 dilution in PBS-BSA 0.1% at 4°C overnight, followed by Alexa fluor 647-conjugated goat anti-mouse IgG in PBS-BSA 0.1% for 1 h at room temperature, and Hoechst 33342.

To determine the positioning of PODXL in Raji cells contacting with T cells, images were acquired in a Zeiss Axio Observer.Z1 microscope equipped with a Plan-Apochromat 20x (0.8) objective, motorized stage, HXP mercury lamp for fluorescence excitation, halogen lamp for DIC imaging, and an Axiocam 503 monochrome digital camera. Zeiss-supplied Zen software controlled illumination, optic filters, stage, Z-positioning for stacks generation and image acquisition. Wavelengths (in nm) of filter sets for GFP fluorescence imaging were, excitation: BP450-490, dichroic: 495 and emission: BP500-550; for Alexa Fluor-555 were, excitation: BP538-562, dichroic: 570 and emission: BP570-640, and for Hoechst 33342 were, excitation: BP335-383, dichroic: 395 and emission: BP420-470. Light was collected in a 1936 x 1460 pixels camera chip at 14 bit/pixel achieving a final resolution of 0.45 µm/pixel. In each field of view, first a DIC and individual fluorescence images of the three fluorophores were captured. The position of PODXL in conjugates comprising one Raji cell and one CD4^+^ T cell was scored as 1 when redistributed close to the contact area, 2 and 3 when located at the intermediate zone, and 4 when presented distally. When PODXL was found in more than one quadrant, the total intensity of GFP of each quadrant was measured using Fiji software (Image J), and the quadrant with the highest fluorescence intensity of GFP determined PODXL-GFP polarization score.

Confocal image-stacks were scanned in a Zeiss LSM 880 Airyscan microscope using 405 (Hoechst), 488 (GFP and Cy2), 514 (Alexa fluor 555), and 633 (Alexa fluor 647) nm laser lines as needed. Optics consisted on a Plan-Apochromat 63x (1.4) objective and its respective main dichroic beam splitters (MBS-405, MBS-488, MBS-458/514, MBS-458/561, MBS-488/561/633). Fluorescence was collected through independent channels with 1 Airy unit adjusted pinhole aperture and emission windows of 425-480 nm, 495-560 nm, 545-695 nm and 640-750 nm. An additional channel was enabled for transmitted light-generating DIC images. Optical sections were 2.5x zoomed areas of 0.35 microns Z-steps digitized at 16-bit, achieving a final resolution of 0.05 microns/pixel. Zen software assisted in laser, dichroic, emission window, image size and z-step selection.

Image stacks were inspected to identify Podocalyxin-GFP overlapping with a cell-to-cell contact and these single planes in each stack were selected for further analysis with Fiji software. Pixels including the cell-to-cell contact were delineated with a polygonal Region-of-Interest (ROI). ROIs were cropped from the optical sections of Alexa 555 red emission and GFP green emission. Pairs of cropped images including the cell-to-cell contact were used for the colocalization analysis provided by the Pearson’s coefficient calculation included in the JACoP plugin (https://imagej.net/plugins/jacop).

### Statistical Analysis

The statistical analyses and graphs were performed using GraphPad Prism 8.0 software. For the analysis of parametric data, paired/unpaired t-test was used when comparing 2 groups, and one/two-way ANOVA was used when comparing more than 2 groups. For the comparison of nonparametric data groups, Wilcoxon test was used for 2 groups and Krustal-Wallis test was used for 3 or more groups. Results are presented as means ± SD. P-values lower than 0.05 are considered significant and represented with the following symbols: *p<0.05, **p<0.01, ***p<0.001 and ****p<0.0001.

## Results

### PODXL Is Expressed by Human Immature Monocyte-Derived DCs

**Figure 1:**
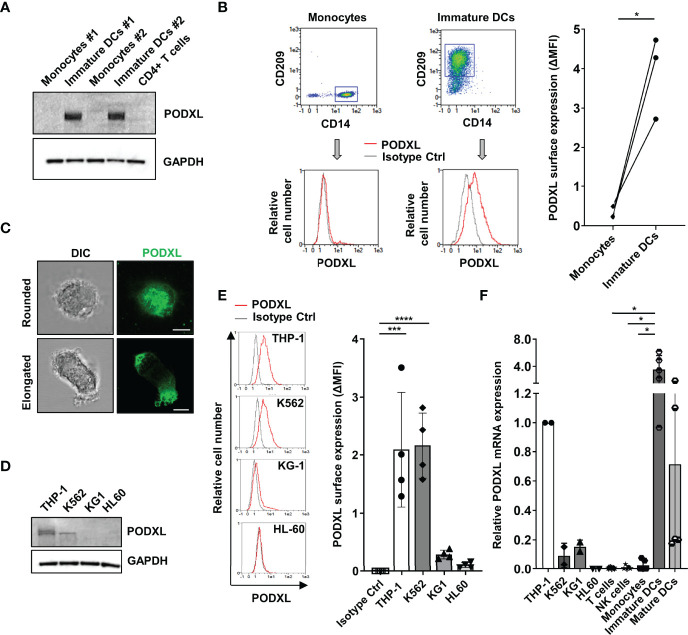
PODXL is expressed in human monocyte-derived immature DCs. **(A)** Determination of PODXL expression in total lysates from human monocytes and monocyte-derived immature DCs from two donors and CD4^+^ T cells from one donor by Western blot analysis using a specific mouse anti-human PODXL monoclonal antibody. GAPDH was used as loading control. The blot shown is representative of two independent experiments. **(B)** Flow cytometry gating and overlay histograms depicting the surface expression of PODXL on monocytes (CD14^+^ CD209^-^) and immature DCs (CD14^-^ CD209^+^) detected by flow cytometry using a biotinylated goat anti-human PODXL antibody (red line) or an isotype control (grey line) followed by PE-conjugated streptavidin from one representative donor. The graph shows PODXL surface expression (**Δ**MFI= PODXL MFI - isotype control MFI) on monocytes and immature DCs from three donors. *p < 0.05, paired t test. **(C)** Expression of PODXL in monocyte-derived immature DCs by fluorescence microscopy. Monocyte-derived immature DCs were deposited onto glass slides using a cytospin and incubated in complete culture medium for 30 min. Cell were stained with a goat anti-PODXL polyclonal antibody followed by an anti-goat Cy2-labelled secondary antibody (green). Images were acquired with a 63x objective using a confocal fluorescence microscope and are representative of ten rounded and twelve elongated immature DCs photographed from three independent experiments. Example shows maximal intensity z-projections of confocal fluorescence sections. DIC, differential interference contrast. Scale bar corresponds to 5μm. **(D)** Determination of PODXL expression in cell lysates from different myelomonocytic cell lines by Western blot analysis using a specific anti-PODXL monoclonal antibody. GAPDH was used as loading control. The blot shown is representative of two independent experiments. **(E)** Overlay histograms showing the surface expression of PODXL on four different myeloid cell lines determined by flow cytometry using a biotinylated goat anti-human PODXL antibody (red line) or an isotype control (grey line) followed by PE-conjugated streptavidin from one representative experiment. Graph shows mean ± SD of PODXL surface expression (**Δ**MFI= PODXL MFI - isotype control MFI) from four independent experiments. ***p < 0.001, ****p < 0.0001, one-way ANOVA. **(F)** Analysis of PODXL mRNA level by RT-PCR in four myelomonocytic cell lines and in T cells, NK cells, monocytes, monocyte-derived immature and mature DCs. Results are depicted relative to THP-1 cells. *p < 0.05, Krustal-Wallis test.

We recently detected upregulated expression of PODXL in breast cancer cells exposed to IL-4 (35). Given that IL-4, in combination with GM-CSF, is routinely used to differentiate blood monocyte into immature DCs *in vitro* (36), we asked whether immature monocyte-derived DCs express PODXL. Hence, we examined the expression of PODXL in whole lysate from human monocyte-derived DCs by Western-blot using a specific anti-human PODXL monoclonal antibody. The results showed a complete absence of PODXL expression in human monocytes as well as in CD4^+^ T cells. On the contrary, a strong band of approximately 160 kDa corresponding to PODXL was detectable in immature DC derived from monocytes incubated with IL-4 and GM-CSF (**Figure 1A**). We next aimed to determine the expression of PODXL on the surface of immature DCs by flow cytometry. In support of the results obtained by Western blot, flow cytometry analysis revealed the expression of PODXL on monocyte-derived immature DCs (CD14^-^ CD209^+^ cells) and the complete absence of this protein on monocytes (CD14^+^ CD209^-^ cells) (**Figure 1B**). The expression of PODXL in immature DCs was also confirmed by confocal fluorescence microscopy in cells displaying both rounded and elongated morphologies (**Figure 1C**). These results indicate that the differentiation of monocytes into immature DCs induces total and cell surface expression of PODXL.

As the myeloid cell line K562 has been reported to express PODXL (30), we determined its presence in a variety of myelomonocytic cell lines. Western blot analysis revealed higher level of PODXL expression in THP-1 cells compared to K562 cells, whereas KG1 and HL-60 cells were negative for PODXL expression (**Figure 1D**). Correspondingly, by flow cytometry analysis, we detected moderate level of PODXL on the surface of THP-1 and K562 and lack of expression on KG-1 and HL60 cells (**Figure 1E**).

In agreement with the results obtained at protein level, qRT-PCR analysis showed that PODXL mRNA was nearly undetectable in monocytes, T cells and NK cells compared to THP-1 cells (**Figure 1F**). In contrast, immature DCs differentiated from monocytes expressed high level of PODXL mRNA, indicating that PODXL expression is transcriptionally upregulated in these cells (**Figure 1F**). Interestingly, PODXL mRNA level decayed after the induction of DC maturation with LPS, a TLR4 agonist broadly used to induce DC maturation *in vitro*. Regarding myeloid cell lines, the levels of PODXL mRNA were notably lower in K562 and KG-1 cells and undetectable in HL-60 cells compared to those observed in THP-1 cells (**Figure 1F**). The presence of PODXL protein in K562 cells and its absence in KG1 cells, despite expressing equally low levels of PODXL mRNA expression, indicate a post-transcriptional regulation of PODXL expression in these cell lines.

### PODXL Is Downregulated in Mature DCs

**Figure 2:**
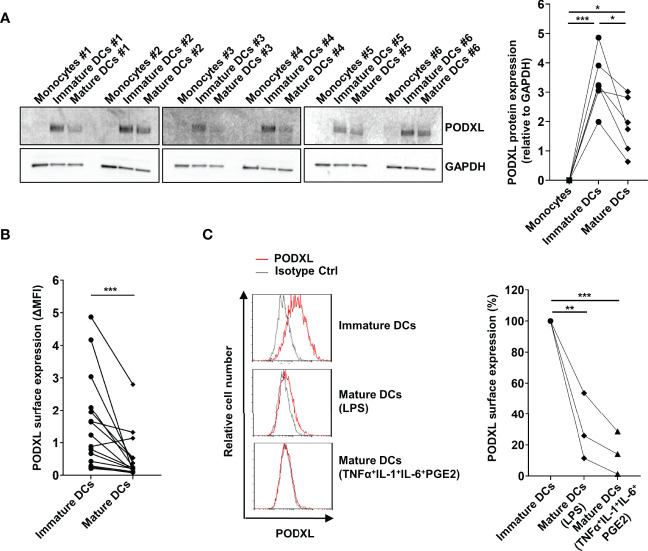
PODXL expression on monocyte-derived DCs is downregulated upon maturation stimuli. **(A)** Determination of PODXL expression in monocytes, monocyte-derived immature and mature DCs in total cell lysates from six donors by Western blot using a specific mouse anti-human PODXL monoclonal antibody. Monocytes were incubated with IL-4 and GM-CSF for 5 days to obtained immature DCs, followed by incubation with LPS for 2 days to induce DC maturation. GAPDH was used as loading control. The bar graph represents the mean ± SD of PODXL levels relative to GAPDH from the six donors of three independent experiments. *p < 0.05, ***p < 0.001, RM one-way ANOVA. **(B)** Expression levels of PODXL on the surface of immature and mature DCs from 15 healthy donors using a mouse anti-human PODXL monoclonal antibody followed by PE-conjugated anti-mouse IgG secondary antibody determined by flow cytometry. Graph shows PODXL surface expression (**Δ**MFI= PODXL MFI - isotype control MFI). ***p < 0.001, Wilcoxon matched-pairs signed rank test. **(C)** Mature DCs were obtained by incubating immature DCs with LPS or the maturation cocktail consisting of TNFα, IL-1, IL-6 and PGE2, and PODXL expression levels determined by flow cytometry as in **(B)**. Overlay histograms show surface expression of PODXL (red line) and isotype control (grey line) from one representative donor. The graph represents PODXL expression relative to immature DCs from three donors in three independent experiments. **p < 0.01, ***p < 0.001, one-way ANOVA.

In order to evaluate the effect of maturation stimuli on PODXL expression at protein level, monocyte-derived immature DCs from six donors were incubated for 2 days in the presence of LPS. Total cell lysates were subjected to Western blot analysis for the detection of PODXL, using GAPDH as control. For all donors analyzed, PODXL expression level was markedly upregulated after DC differentiation from monocytes, as shown above, and exhibits donor-to-donor heterogeneity. However, upon LPS-maturating stimulus, PODXL expression was notably downregulated, although at different degrees among donors (**Figure 2A**). In agreement with these results, surface expression of PODXL on DCs was greatly decreased in response to LPS in almost all the donors analyzed (**Figure 2B**). To figure out whether this reduction in PODXL expression was exclusively induced by LPS, we tested PODXL expression on DCs using different maturation stimuli. When DCs were stimulated with the standard maturation cocktail consisting of TNF-α, IL-1β and IL-6 cytokines and prostaglandin E2, the surface expression of PODXL was strikingly diminished (**Figure 2C**). These data indicate that PODXL expression in DCs is negatively regulated by diverse maturation-inducing stimuli.

### PODXL Expression in Myeloid Cells Is Positively Regulated by IL-4 Through MEK/ERK and JAK3/STAT6 Signaling Pathways

**Figure 3:**
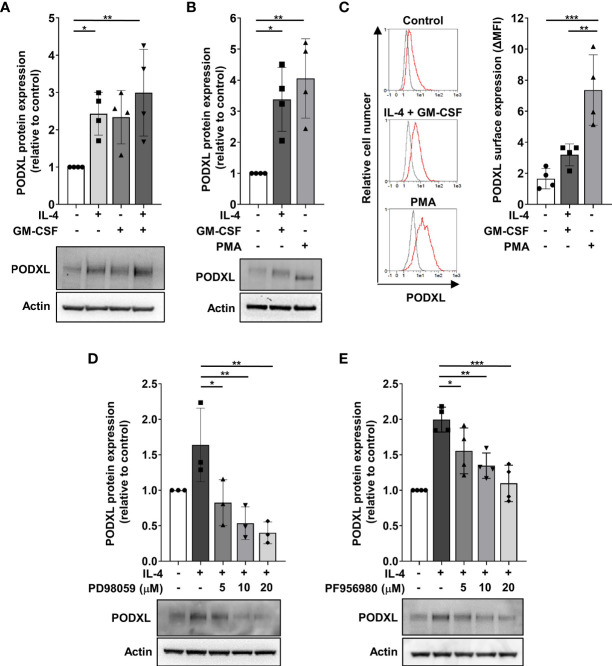
PODXL expression is positively regulated by IL-4 through MEK/ERK and JAK3/STAT6 signaling pathways in myeloid cells. THP-1 cells were stimulated with IL-4 (400 U/ml) and GM-CSF (800 U/ml) cytokines **(A)** for 96 h or with PMA (100 ng/ml) **(B)** for 48 h followed by a resting period of 48 (h) Afterwards, total cell lysates were subjected to Western blotting using a specific monoclonal antibody against PODXL. Actin was used as loading control. Bar graphs represent the mean ± SD of PODXL levels relative to those of unstimulated cells of four independent experiments. *p < 0.05, **p < 0.01, one-way ANOVA. **(C)** PODXL surface expression was analyzed by flow cytometry using a biotinylated goat anti-human PODXL antibody followed by PE-conjugated streptavidin. Overlay histograms show surface expression of PODXL (red line) and isotype control (grey line) from one representative donor. Graph shows PODXL surface expression (ΔMFI= PODXL MFI - isotype control MFI) of four independent experiments. **p < 0.01, ***p < 0.001, one-way ANOVA. **(D, E)** Effect of signaling pathway inhibitors on IL-4-induced PODXL expression in THP-1 cells. Cells were incubated with 400 U/ml IL-4 in the presence of increasing concentration of MEK/ERK signaling pathway inhibitor (PD98059) **(D)** or JAK3 signaling pathway inhibitor (PF956980) **(E)** for 96 (h) Then, cell lysates were subjected to Western blot analysis with a monoclonal anti-PODXL antibody. Actin was used as loading control. Bar graphs represent the mean ± SD of PODXL levels relative to those of unstimulated and untreated cells of three **(D)** or four **(E)** independent experiments. A representative blot is shown below each graph. *p < 0.05, **p < 0.01, ***p < 0.001, one-way ANOVA.

To get insight into the molecular mechanism underlying cytokine-induced PODXL expression in myeloid cells, we used THP-1 cell line as a model for studying DC and macrophage differentiation, as previously described (38, 39). The expression of DC markers in THP-1 cells stimulated with IL-4 and GM-CSF and of macrophage markers in cells treated with PMA to model macrophage differentiation was checked by flow cytometry (**Supplementary Figure S1**). Furthermore, the morphological characteristics of DCs and macrophages in these cells were examined using light microscopy (**Supplementary Figure S1**). First, we analyzed whether PODXL could be up-regulated in THP-1 cell line in response to IL-4 and GM-CSF by Western blot. The results revealed that these cytokines equally increased the expression of PODXL, although no additive effect was detected in response to the combination of both cytokines (**Figure 3A**). When THP-1 cells were stimulated with PMA, Western blot revealed a band corresponding to PODXL with a molecular weight lower than that observed in THP-1 cells stimulated with IL-4 and GM-CSF, suggesting a differential expression of PODXL glycoforms in macrophages and DCs (**Figure 3B**). Flow cytometry analysis showed that PODXL expression augmented on THP-1 cell surface in response to the combination of IL-4 and GM-CSF and, to a significantly greater extent, by PMA stimulation (**Figure 3C**).

Binding of IL-4 to its receptor activates the Janus kinase 3 (JAK3)/signal transducer and activator of transcription (STAT) 6 pathway and MEK/ERK pathway, triggering the transcription of IL-4-inducible genes (40, 41). To examine whether these signaling pathways are involved in IL-4-induced PODXL expression in myeloid cells, we stimulated THP-1 cells with IL-4 in the presence of increasing concentrations of specific inhibitors of MEK/ERK (PD98059) and JAK3/STAT6 (PF956980) pathways, and the expression of PODXL was detected by Western blot. The results showed that treatment of THP-1 cells with PD098059 markedly decreased IL-4-mediated as well as basal expression of PODXL in a dose-dependent manner (**Figure 3D**). IL-4-induced PODXL expression was also dose-dependently reduced following PF956980 treatment (**Figure 3E**). These results indicate that IL-4 induces PODXL expression in THP-1 myeloid cells through MEK/ERK and JAK3/STAT6 signaling pathways.

### PODXL Expressed by APCs Enhances APC-T Cell Interaction

**Figure 4:**
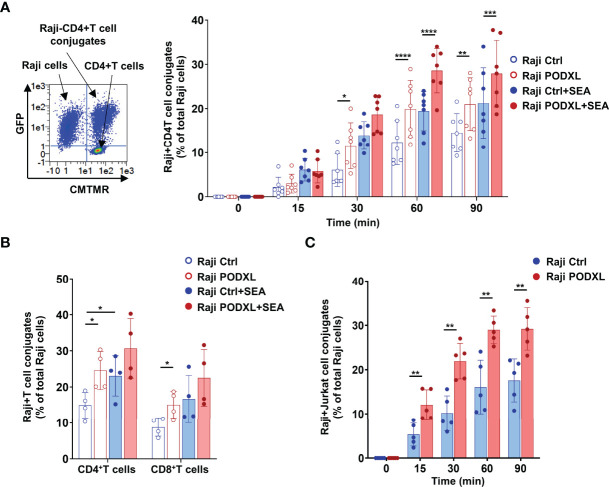
PODXL expressed in APCs enhances both APC-CD4^+^ T cell and APC-CD8^+^ T cell interactions. **(A)** Raji cells overexpressing PODXL (red) or Raji-Ctrl cells (blue) were pulsed or not with SEA. Afterwards, CD4^+^ T cells isolated from PBMCs and pre-labelled with CMTMR were mixed with Raji-PODXL or Raji-Ctrl at a ratio of 1:1 and incubated for different time points at 37°C. The formation of conjugates between Raji cells (GFP^+^) and CD4^+^ T cells (CMTMR^+^) was detected as CMTMR^+^ and GFP^+^ events by flow cytometry. A representative density plot out of seven independent experiments is shown. Graph shows mean ± SD of the percentage of conjugates (CMTMR^+^GFP^+^) from the total Raji cells (GFP^+^) of four independent experiments. *p < 0.05, **p < 0.01, ***p <0.001, ****p < 0.0001, two-way ANOVA. **(B)** Raji-PODXL or Raji-Ctrl cells were mixed with isolated CD4^+^ T and CD8^+^ T at a ratio of 1:1 and incubated for 30 min. CD4^+^ and CD8^+^ T cells were detected using anti-CD4-APCH7 and anti-CD8-APCH7 monoclonal antibodies by flow cytometry. The percentage of Raji-CD4^+^ T cell (GFP^+^APCH7^+^) or Raji-CD8^+^ T cell (GFP^+^APCH7^+^) out of the total Raji cells was determined. Bar graph represents the mean ± SD of five independent experiments. *p < 0.05, RM one-way ANOVA. **(C)** Raji-PODXL or Raji-Ctrl cells were mixed with CMTMR-labeled Jurkat cells at a ratio of 1:1 and incubated at different time points. The percentage of Raji-Jurkat cell conjugates was determined by flow cytometry as in **(A)**. Bar graph represents the mean ± SD of five independent experiments. **p < 0.01, two-way ANOVA.

Given that PODXL has previously been reported to promote intercellular adhesion (34), we sought to determine whether PODXL expressed by APCs participates in APC-T cell interaction. To address this possibility, Raji cells overexpressing PODXL-GFP (Raji-PODXL) or Raji control (Raji-Ctrl) cells were preincubated with or without SEA superantigen and mixed with isolated CD4^+^ T cells labelled with the red dye CMTMR at a ratio of 1:1 (**Figure 4A**). After incubation for different periods of time, cells were analyzed by flow cytometry to quantify double-positive events corresponding to Raji-CD4^+^ T cell conjugates. The results showed that Raji-PODXL cells formed significantly more conjugates than Raji-Ctrl cells in a time dependent manner, reaching the highest level at 60 min of incubation, both in the presence and in the absence of SEA (**Figure 4A**). However, SEA-induced conjugate formation was slightly lower in Raji-PODXL cells compared to Raji-Ctrl cells at 30 min of incubation (**Supplementary Figure 2**). These data indicate that PODXL increases antigen-independent but reduces SEA-induced Raji-CD4^+^ T cell interaction.

To further explore whether the enhanced effect of PODXL on Raji-CD4^+^ T cell conjugate formation also applies to Raji-CD8^+^ T cell conjugates, we next incubated Raji-PODXL cells or Raji-Ctrl cells with total PBMCs, which contains both CD4^+^ T and CD8^+^ T cells. As expected, flow cytometry analysis revealed that Raji-PODXL cells formed more conjugates with CD4^+^ T cells, compared with Raji-Ctrl cells. Nevertheless, we observed no differences between Raji-PODXL and Raji-Ctrl cells in conjugate formation with CD8^+^ T cells (**Supplementary Figure 3**). To exclude a potential competition between CD4^+^ T cells and CD8^+^ T cells for their interaction with Raji-PODXL cells that could abrogate PODXL-induced Raji-PODXL-CD8^+^ T cell conjugate formation, we performed the above experiment using isolated CD4^+^ T and CD8^+^ T cells. The data showed that Raji-PODXL cells formed more conjugates with both isolated CD4^+^ T cells and, to a lesser extent, CD8^+^ T cells than Raji-Ctrl cells **Figure 4B**). We also detected an increased conjugate formation with Raji-PODXL cells compared to Raji-Ctrl cells when Jurkat cells were used as CD4^+^ T cells (**Figure 4C**). Hence, these data indicates that PODXL enhances both APC-CD4^+^ T cells and APC-CD8^+^ T cell interactions.

### PODXL Reduces CD86, MHC-I and MHC-II Expression in APCs

**Figure 5:**
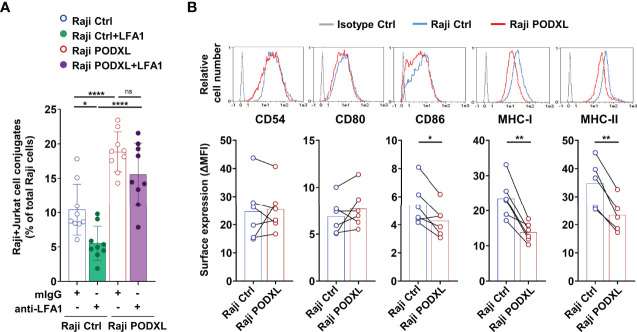
PODXL decreases the expression of CD86, MHC-I and MHC-II in APCs. **(A)** Raji-PODXL, Raji-Ctrl cells and CMTMR-labeled Jurkat cells were preincubated separately with a blocking anti-LFA-1 monoclonal antibody for 15 min. Afterwards, Raji-PODXL and Raji-Ctrl were mixed with CMTMR-labeled Jurkat cells at a ratio of 1:1 and incubated for 30 min. The percentage of Raji-Jurkat cell conjugates was determined by flow cytometry as in (**Figure 4A**). Bar graph represents the mean ± SD of nine independent experiments. *p < 0.05, ****p < 0.0001, one-way ANOVA. **(B)** Raji cells overexpressing PODXL and Raji Ctrl cells were analyzed by flow cytometry for surface expression of CD54, CD80, CD86, MHC-I and MHC-II. Overlay histograms represent the surface expression of the indicated marker in Raji-PODXL cells (red line), Raji-Ctrl cells (blue line) and the isotype control (grey line). Graphs show mean ± SD of surface expression (ΔMFI= Marker MFI - isotype control MFI) from six (CD54, CD80, CD86, MHC-I) or five (MHC-II) independent experiments. *p < 0.05, **p < 0.01, paired t test. 0.0001, ns, not statistically significant; one-way ANOVA.

The binding of integrin lymphocyte function antigen-1 (LFA-1) expressed on T cells to ICAM-1 (CD54) displayed on APCs is crucial for stabilizing the APC-T cell interaction during immune synapse assembly (42, 43). To establish whether LFA-1 mediates PODXL-induced APC-CD4^+^ T cell adhesion, Raji-PODXL or Raji control cells and Jurkat cells were pretreated with a blocking antibody against LFA-1 or an isotype control and then incubated together for 30 min in the presence of the antibody. The results showed a decreased formation of Raji-Ctrl-CD4^+^ T cell conjugates but not significant reduction in the number of Raji-PODXL-CD4^+^ T cell conjugates after treatment with the anti-LFA-1 antibody (**Figure 5A**). These findings suggest that PODXL-induced APC-T cell interaction is independent on LFA-1.

We next asked whether PODXL could regulate the expression of CD54 and other molecules expressed in APCs that participate in APC-T cell conjugate stabilization. The determination of CD54 expression on Raji-PODXL and Raji-Ctrl cells by flow cytometry yielded similar values (**Figure 5B**). Among the analyzed molecules, the results revealed similar levels of CD80 and significantly lower expression of CD86, MHC-I and MHC-II in Raji cells overexpressing PODXL compared to Raji-Ctrl cells (**Figure 5B**). These data indicate that PODXL reduces the expression of CD86, MHC-I and MHC-II on the surface of Raji cells.

### PODXL Polarizes Toward the APC-CD4^+^ T Cell Contact Site

**Figure 6:**
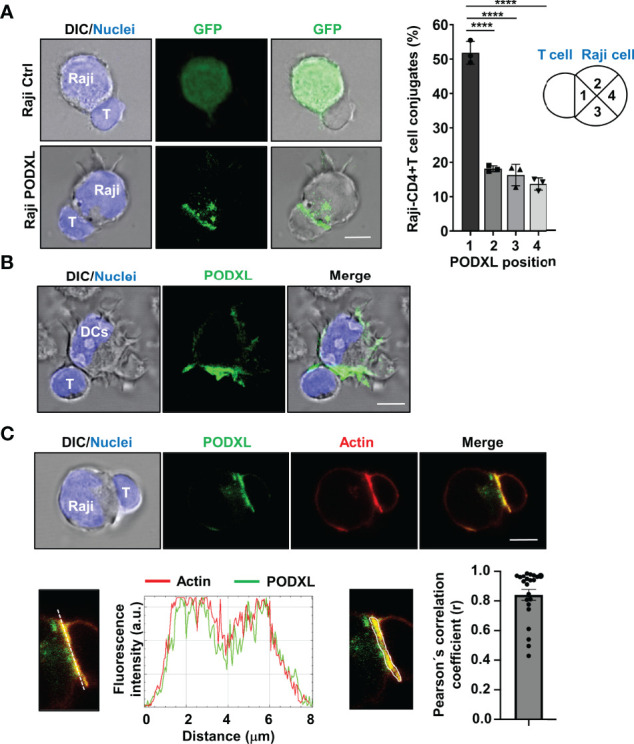
PODXL expressed in APCs localizes to the T-cell contact side and partially colocalizes with actin. **(A)** Raji cells overexpressing PODXL-GFP and pulsed with SEA were incubated with CD4^+^ T cells at a ratio of 1:2 for 30 min. Images of conjugates comprising one Raji cell and one CD4^+^ T cell were obtained using confocal fluorescence microscopy with a 63x objective and are representative of three independent experiments. The example shows maximal intensity z-projections of confocal fluorescence sections. The position of PODXL in Raji-CD4^+^ T cell conjugates was determined using images obtained with a fluorescence microscope and a 20x objective and scored as 1 when located in the quadrant close to the contact area, 2 and 3 when located at the intermediate quadrants, and 4 when present at the distal quadrant. Bar graph represents the percentage of conjugates with PODXL in each indicated position from three independent experiments and a total of 426 Raji-CD4^+^ T cell conjugates. The results are shown as mean ± SD. ****p < 0.0001, one-way ANOVA. **(B)** Immature DC were incubated with CD4^+^ T cells for 30 min and conjugates were analyzed with a 63x objective using a confocal fluorescence microscope. Cells were stained with a goat polyclonal anti-PODXL antibody followed by an anti-goat Cy2-labelled secondary antibody (green), and Hoechst 33342 for nuclei staining (blue). Images show a representative immature DC-CD4^+^ T cell conjugate of 15 conjugates photographed from three independent experiments. Example shows maximal intensity z-projections of confocal fluorescence sections. **(C)** Raji cells overexpressing PODXL-GFP (green) and pulsed with SEA were incubated with CD4^+^ T cells and stained for actin with Alexa 555-phalloidin (red) and for nuclei with Hoechst 33342 (blue). A confocal single z-section from a representative conjugate is depicted. An enlarged image of the region corresponding to the cell-to-cell contact zone is shown. Histogram depicts intensity profiles of PODXL (green) and actin (red) obtained using ImageJ-Fiji software, along an ideal line (white dashed line on the left image) crossing the contact zone of a representative conjugate. Bar graph represent the Pearson´s correlation coefficient of PODXL and actin colocalization at cell-to-cell contact site in Raji-PODXL cells conjugated with CD4^+^ T cells. A representative image showing the ROI selected for the quantification of colocalization is depicted (right image). Twenty two Raji-PODXL- CD4^+^ T cell conjugates from five independent experiments were analyzed. Error bar displays the standard error. Scale bars in confocalimages correspond to 5 um.

We previously reported that PODXL overexpressed in MCF-7 breast cancer cell line accumulates at the interface formed with NK cells (35). To explore whether PODXL expressed in APCs is recruited toward the contact area formed with CD4^+^ T cells, Raji cells overexpressing PODXL-GFP were incubated with CD4^+^ T cells for 60 min. Then, the position of PODXL was determined microscopically in conjugates comprising one Raji cell and one CD4^+^ T cell, and scored as 1 when redistributed close to the contact area, 2 and 3 when located at the intermediate zone, and 4 when presented distally (**Figure 6A**). The results showed that PODXL localized proximal to the interface in approximately 50% of the conjugates, doubling the percentage expected for a random distribution of the protein (**Figure 6A**). On the contrary, PODXL redistributed to 2 or 3 intermediate positions in 18% and 16% of the conjugates, respectively, and to a distal position only in 13% of the cases (**Figure 6A**). Then, we aimed to determine the location of PODXL in human immature monocyte-derived DCs conjugated with CD4^+^ T cell by immunostaining with a specific antibody against human PODXL and confocal microscopy analysis. The results showed that PODXL localizes at the DC-CD4^+^ T cell contact site (**Figure 6B**). Altogether, these results demonstrate that PODXL expressed in APC cells polarize at the contact zone formed with CD4^+^ T cells.

When an APC encounters a T cell, F-actin redistributes to the APC-T cell interface forming a highly dynamic structure known as immune synapse (16, 17). Given that PODXL binds to F-actin in various cell types, we next asked whether PODXL colocalizes with F-actin at the APC-T cell contact zone. Using Raji cells overexpressing PODXL-GFP, we observed that PODXL partially colocalized with the actin cytoskeleton at the contact area formed between Raji cells and CD4^+^ T cells (**Figure 6C**).

### PODXL Expressed by APCs Alters the Translocation of CD4^+^ T Cell Centrosome Toward the Contact Site

**Figure 7:**
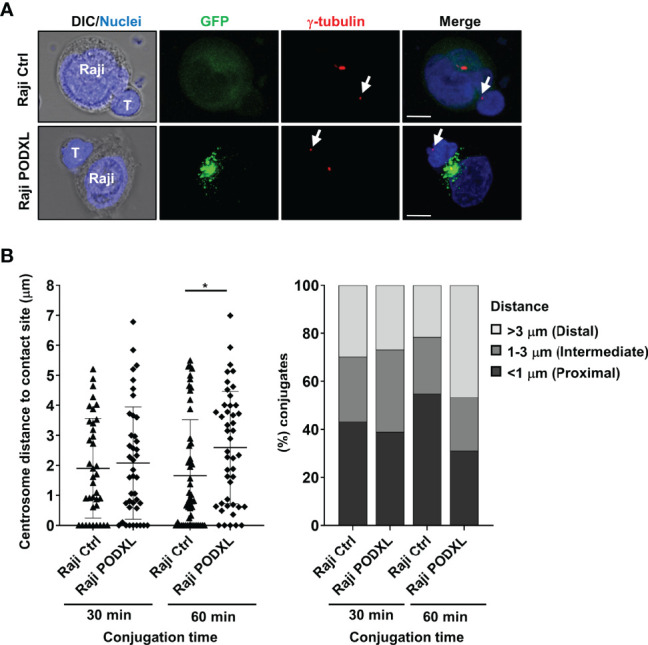
PODXL expressed in APCs perturbs CD4^+^ T cell-centrosome polarization to the contact site. **(A)** Raji PODXL or Raji control cells pulsed with SEA were incubated with CD4^+^ T cells at a ratio of 1:1 for 30 min or 60 min. Then, cells were stained with a monoclonal antibody against γ-tubulin to visualize the centrosome (red) and Hoechst 33342 for nuclei staining (blue). Confocal fluorescence microscopy images show separate and merged channels of maximal intensity z-projections of z-sections from a representative experiment performed at 60 min of incubation. Arrows point to T-cell centrosome in each T-cell. DIC: differential interference contrast. Scale bars correspond to 5 μm. **(B)** Quantification of T-cell centrosome polarization in Raji-CD4^+^ T cells conjugates. Scatter graph depicts the distance of CD4^+^ T cell centrosome to Raji-cell contact site in each conjugate for a total of 41 (Raji Ctrl, 30 min), 37 (Raji PODXL, 30 min), 51 (Raji Ctrl, 60 min), and 45 (Raji PODXL, 60 min) conjugates from three independent experiments. The results are shown as mean ± SD. *p < 0.05, two-way ANOVA followed by Holm-Šídák multiple comparisons test. Bar graph represents the percentage of conjugates in which T-cell centrosome localizes at a distal (**≥**3 μm), intermediate (1-3 μm) or proximal (**≤**1 μm) distance from Raji-cell contact site for the indicated conditions.

Upon APC and CD4^+^ T cell conjugate formation, T cell centrosome polarizes toward the contact side, allowing the reorientation of the T cell secretory organelles toward the APC and the directional secretion of cytokines to favor T cell effector functions (19, 20). To evaluate the effect of PODXL expressed by APCs in the translocation of CD4^+^ T cells to the contact site, Raji-PODXL cells and Raji-Ctrl cells were incubated with CD4^+^ T cells for 30 min and 60 min. Cells were then immunostained with γ-tubulin to visualize the centrosome by confocal microscopy and the distance of CD4^+^ T cell centrosomes to APC contact site was analyzed (**Figure 7A**). At 30 min of incubation, there were no differences in CD4^+^ T cell centrosome distance to the interface of conjugates formed with Raji-PODXL compared to Raji-Ctrl cells (**Figure 7B**). However, at 60 min, the centrosome distance to the contact site in CD4^+^ T cells interacting with Raji-PODXL cells was significantly higher than that observed in CD4^+^ T cells contacting with Raji-Ctrl cells (**Figure 7B**). Correspondingly, the percentage of Raji-PODXL-CD4^+^ T cell conjugates with centrosome located at a distance lower than 1 μm was lower than that observed in Raji-Ctrl-CD4^+^ T cell conjugates at 60 min of incubation (**Figure 7B**). These findings point to a role of PODXL in regulating centrosome polarization in CD4^+^ T cell upon interaction with APCs.

## Discussion

In this work, we identify PODXL as a protein expressed by human monocyte-derived immature DCs that undergoes downregulation in response to inflammatory stimuli. To our knowledge, this is the first study reporting the expression of PODXL in DCs. Our results showed that the decrease of PODXL expression in DCs occurred in response to various molecules broadly used for DC maturation *in vitro*, such as LPS and a cocktail containing TNFα, IL-1β, IL-6 and PGE2 (44, 45). LPS is a major inflammatory component of gram-negative bacteria that binds to TLR4 and triggers the release of proinflammatory cytokines, the upregulation of costimulatory molecules, and the activation of antigen presentation on APCs, inducing both innate and adaptive immune responses (44). On the other hand, the cocktail containing TNFα, IL-1β, IL-6 and PGE2 has been described to induce Th1-polarized immune responses (46). These observations suggest that PODXL could exert tolerogenic or anti-inflammatory effects in immature DCs, so that its downregulation in response to inflammatory stimuli would favor an effective immune response. Moreover, our data revealed highly heterogeneous levels of PODXL expression in immature monocyte-derived DCs as well as a varied degree of PODXL downregulation in mature DCs among donors. The heterogeneity of PODXL expression levels in immature DCs might be explained by genetic or epigenetic variations in PODXL gene between donors. In line with this suggestion, polymorphisms in the promoter region of DC-SIGN have been shown to influence the expression level of this gene in immature DCs (47). Part of the variability in PODXL expression could also depend on basal inflammatory state, age or gender of individuals, since these variables influence the transcriptional profile of DCs (48). Our results further demonstrates that PODXL expressed on APCs perturbs CD4^+^ T cell centrosome translocation to the contact site, a process required for proper T-cell effector function (19). These findings suggest that the capacity of DCs to downregulate the level of PODXL expression in response to inflammatory stimuli might modulate the strength of the immune response.

The extracellular domain of PODXL displays extensive N- and O-glycosylation and sialylation, including alpha 2,6-sialylation (28, 49). Immature monocyte-derived and tolerogenic DCs has been demonstrated to present high level of alpha 2,6-sialic acid-containing glycoproteins, which decreases upon DC maturation (50), an expression pattern comparable to that observed for PODXL in our study. Previous studies have attributed a role for sialoglycoproteins expressed by DCs in the modulation of immune responses (50, 51). Alpha 2,6-sialic acid-containing glycans expressed on immature and tolerogenic DC has been reported to promote immune tolerance by binding to inhibitory siglecs expressed on effector T cells (50). Additional studies will be required to determine whether the sialomucin PODXL expressed on DCs regulates T cell responses, including cytokine release, clonal expansion and cell survival.

An earlier study demonstrated that IL-4 induces monocyte differentiation into immature DCs through the demethylation and subsequent expression of DC-specific genes *via* activating JAK3/STAT6 signaling pathway (52). Another report pointed to a role of MEK/ERK signaling pathway in the differentiation and survival of monocyte-derived immature DCs (53). These observations are in line with our data showing increased expression of PODXL in myeloid THP-1 cells and immature DCs derived from monocytes in response to IL-4 or a combination of IL-4 and GM-CSF, respectively. In addition, our results prove the involvement of JAK3/STAT6 as well as MEK/ERK signaling pathways in IL-4-induced PODXL expression in THP-1 cells. The mechanism of PODXL expression in THP-1 cells revealed in our study resembles that of the DC marker CD209 (DC-SIGN; DC-specific ICAM-3-grabbing nonintegrin), whose expression has been reported to depend on JAK/STAT and MEK/ERK signaling pathways in the same cells (41, 54).

Several studies support the role of PODXL in normal and tumor cell adhesion, migration and polarity (28, 55–60). PODXL has been reported to directly interact with or to activate molecules implicated in these processes (33, 61). For instance, PODXL interacts with the actin-binding protein ezrin, which belongs to the ezrin/radixin/moesin (ERM) family (33). ERM proteins regulate the formation of immune synapse by inducing changes in membrane rigidity (62). In line with this, our results showed PODXL polarization to the APC-T cell contact site where it partially colocalizes with actin. Furthermore, PODXL has been shown to activate cell division cycle 42 (Cdc42) (61), a member of the Rho guanosine triphosphatase family of signaling proteins that govern actin organization, migration, adhesion, intercellular trafficking and membrane ruffling as well as cell polarity (63, 64). In DCs, Cdc42 coordinates the formation of the leading edge necessary for cell migration (65) and controls endocytic activity, cell polarity and polarized release of cytokines towards the immune synapse (66, 67). A study stated that Cdc42 GTP, the active form of Cdc42, is expressed only in immature DCs and gradually diminished during DC maturation (66), which agrees with our finding that PODXL is mainly detected in the immature state of DCs and decreases in response to maturation stimuli. Another study reported that Cdc42-mediated actin dynamics maintain DCs in an immature state and the inhibition of Cdc42 activity in mature DCs allows the secretion of proteins and induces the upregulation of the co-stimulatory molecule CD86 (68). Correspondingly, we found that PODXL overexpression in APCs diminishes surface expression of CD86, suggesting a potential regulatory role for PODXL in these processes.

Previous studies have documented the involvement of PODXL in intercellular adhesion (34, 55, 69). Our results indicate that PODXL expressed by APCs increases the interaction with CD4^+^ T and Jurkat cells, as well as with CD8^+^ T cells, suggesting a role for PODXL in the stabilization of APC-CD4^+^ T cell and APC-CD8^+^ T cell interactions. On the contrary, we previously reported a lack of effect of PODXL expressed in MCF-7 breast cancer cells on the formation of MCF-7-NK cells conjugates (35). These discrepancies may reflect a differential expression of PODXL ligands on T cells and NK cells. In early stages of T cell priming, it has been reported that T cells contact with DCs in an antigen-independent manner to scan DCs in search for specific antigen-MHC complexes (70, 71). These interactions are mediated by integrins and other molecules, such as DC-SIGN (71). Once the TCR recognizes the specific antigen-MHC complex, DC-T cell interaction is strengthened through the engagement of LFA-1 with ICAM-1 during the formation of the immune synapse (72). Our results suggest that PODXL-induced APC-T cell interaction is antigen-independent and does not involved the interaction of LFA-1-with ICAM-1. Moreover, we demonstrated that PODXL in APCs polarizes towards T cell contact site, suggesting that PODXL may promote the initial contact between APCs and T cells. Contrary to expected results, we observed downregulated levels of MHC-I, MHC-II and CD86 on PODXL-overexpressing cells. Thus, it is likely that binding of PODXL to its putative ligand on T cells could overcome the negative effect that the reduction of CD86, MHC-I and MHC-II levels by PODXL would exert on APC-T cell interaction. Alternatively, PODXL may alter the expression or function of other adhesion molecules involved in such an interaction.

During APC-T cell interaction, activating signals, including the costimulatory signal delivers by the CD86 ligand CD28, induce T cell centrosome translocation to the immune synapse, facilitating the directional secretion of T-cell cytokines-loaded vesicles toward the contacting APC (19, 20, 73). The altered repositioning of T-cell centrosome in CD4^+^ T cells interacting with Raji-PODXL cells showed in this study could be due to the decreased levels of MHC-II and CD86 on these cells, which would lower TCR and CD28 signal intensity. Centrosome translocation also requires the previous removal of actin from the center of cell-to-cell interface, followed by its accumulation at the periphery to form a mature immune synapse (74). Although most studies addressing the role of actin reorganization have focused their interest on the T cell side of the immune synapse, DC actin depolymerization has been shown to alter immune synapse structure and to compromise the ability of DC to stimulate T cells, highlighting the importance of actin on the DC side of the immune synapse (75). Accordingly, DC cortical stiffness controlled by the actin cytoskeleton has been found to regulate T cell responses (76). Our findings that PODXL reduces T-cell centrosome translocation to the APC-CD4^+^ T cell interface led us to speculate that PODXL could alter the reorganization of the actin cytoskeleton and restrain actin clearance from the center of the contact site, ultimately preventing repositioning of T-cell centrosome and thereby T-cell mediated immune responses. In agreement with this hypothesis, a previous work of our group demonstrated that PODXL expressed on MCF-7 breast cancer cell line accumulates at the immune synapse formed with NK cells and inhibits NK cell activity as well as agonist-induced CD4^+^ T and CD8^+^ T cell proliferation (35).

In conclusion, our data demonstrate that PODXL is expressed in human monocyte-derived immature DCs but is markedly reduced in mature DCs, suggesting that it could serve as a marker of DC maturation state. We also show that PODXL expressed in APCs polarizes to CD4^+^ T cell contact site and enhances APC interaction with CD4^+^ T and CD8^+^ T cells while reduces CD4^+^ T-cell centrosome translocation to the contact site, implying that this protein may negatively modulate T cell activity. Further research will be required to define the functional relevance of PODXL expressed by DCs in the regulation of immune responses and the development of immune-related pathological conditions. The presence of PODXL on the surface of DCs would make this protein a suitable therapeutic target for manipulating the immune response to treat cancer and infectious diseases.

## Data Availability Statement

The raw data supporting the conclusions of this article will be made available by the authors, without undue reservation.

## Ethics Statement

The studies involving human participants were reviewed and approved by Comité Etico de Investigación Clínica (C.E.I.C), Hospital Universitario Cruces. The patients/participants provided their written informed consent to participate in this study.

## Author Contributions

LA and SL conceived and designed the study and wrote the manuscript. LA, JD-G, ET-O and SL performed the experiments and acquired the data. All authors contributed to the interpretation of the data and the revision of the manuscript.

## Funding

This work was supported in part through the grant PI08/1112 and the grant PI18/00629 (Co-funded by European Regional Development Fund; “A way to make Europe”) by Instituto de Salud Carlos III. LA was supported by EITB Maratoia-BIOEF (Basque Foundation for Health Innovation and Research) through the grant BIO11/CM/007. ET-O was recipient of a predoctoral fellowship from the University of the Basque Country EHU/UPV. SL was supported by Miguel Servet I (CP06/00367) and Miguel Servet II research contracts funded by Instituto de Salud Carlos III.

## Conflict of Interest

The authors declare that the research was conducted in the absence of any commercial or financial relationships that could be construed as a potential conflict of interest.

## Publisher’s Note

All claims expressed in this article are solely those of the authors and do not necessarily represent those of their affiliated organizations, or those of the publisher, the editors and the reviewers. Any product that may be evaluated in this article, or claim that may be made by its manufacturer, is not guaranteed or endorsed by the publisher.

## Abbreviations

7-AAD, 7-aminoactinomycin D; APC, antigen presenting cell; BSA, bovine serum albumin; CMTMR, [(4-choromethyl)-benzoyl) amino]-tetramethylrhodamine; DC, dendritic cell; ERM, ezrin/radixin/moesin; GAPDH, glyceraldehyde-3-phophate dehydrogenase; GFP, green fluorescent protein; GM-CSF, granulocyte-macrophage colony-stimulating factor; JAK3/STAT6, Janus kinase 3/signal transducer and activator of transcription 6; ICAM-1, intercellular adhesion molecule-1; LFA-1, lymphocyte function-associated antigen-1; LPS, lipopolysaccharide; MEK/ERK, mitogen-activated protein kinase kinase/extracellular signal-regulated kinase; MFI, median fluorescence intensity; MHC, major histocompatibility complex; PBMC, peripheral blood mononuclear cells; PGE2, prostaglandin E2; PODXL, podocalyxin; SEA, staphylococcal enterotoxin A; TCR, T cell receptor; TNFα, tumor necrosis factor α.
