# Supplementary figures and images for: Podocalyxin Expressed in Antigen Presenting Cells Promotes Interaction With T Cells and Alters Centrosome Translocation to the Contact Site
Source: Front Immunol. 2022 May 31;13:835527. doi: 10.3389/fimmu.2022.835527 (PMC9197222; doi:10.3389/fimmu.2022.835527)

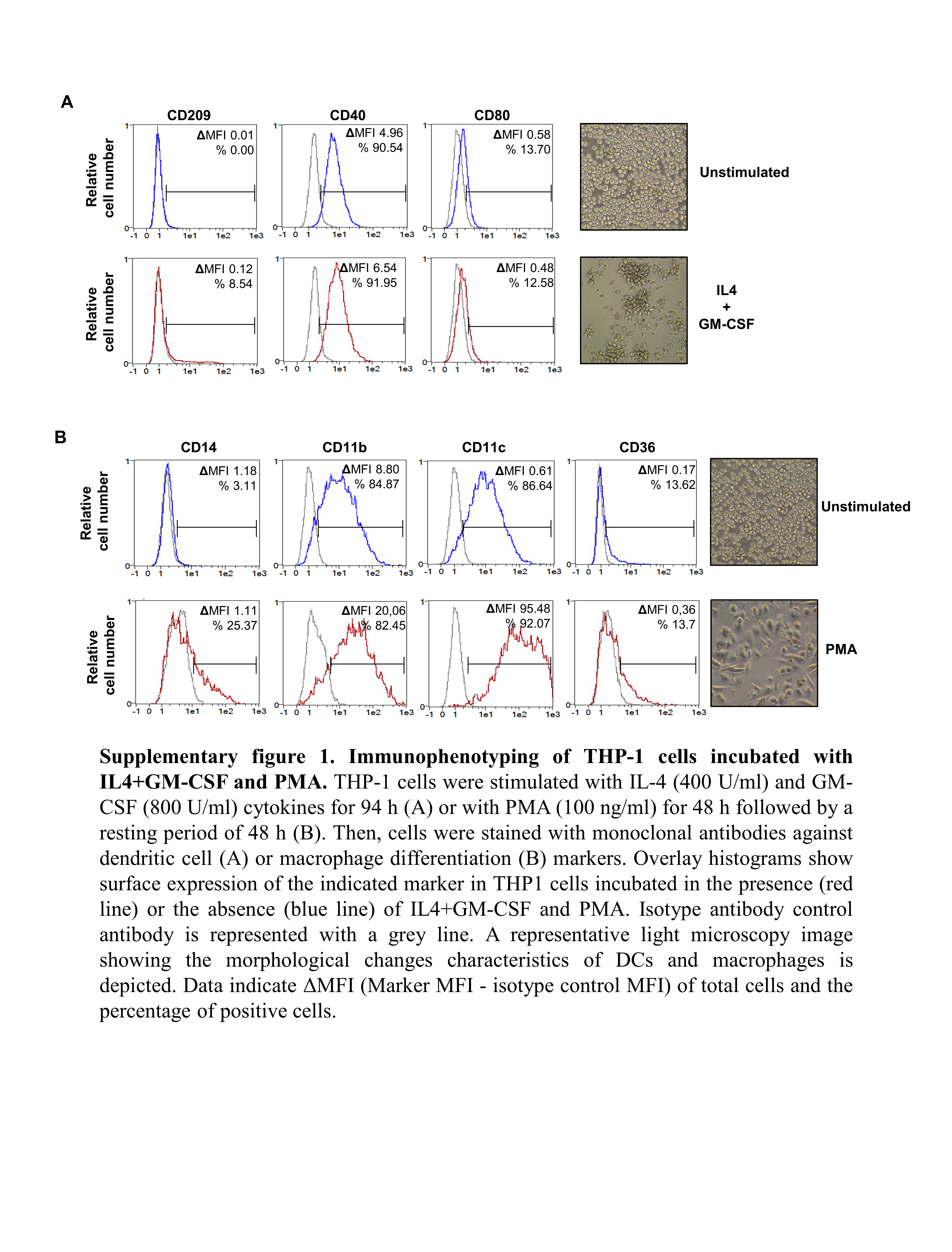

Supplement: Supplementary file 1 [file Image_1.tif]

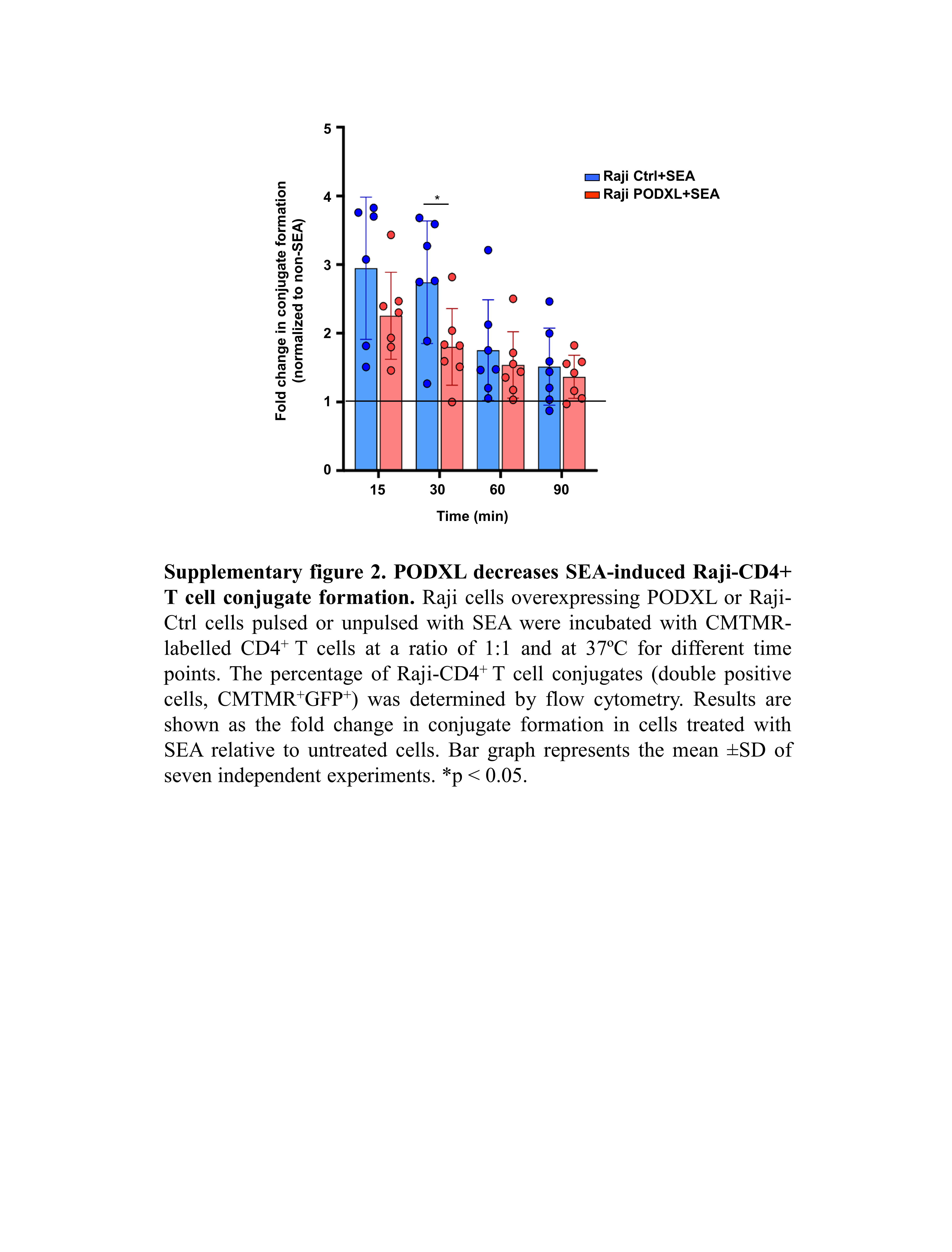

Supplement: Supplementary file 2 [file Image_2.tif]

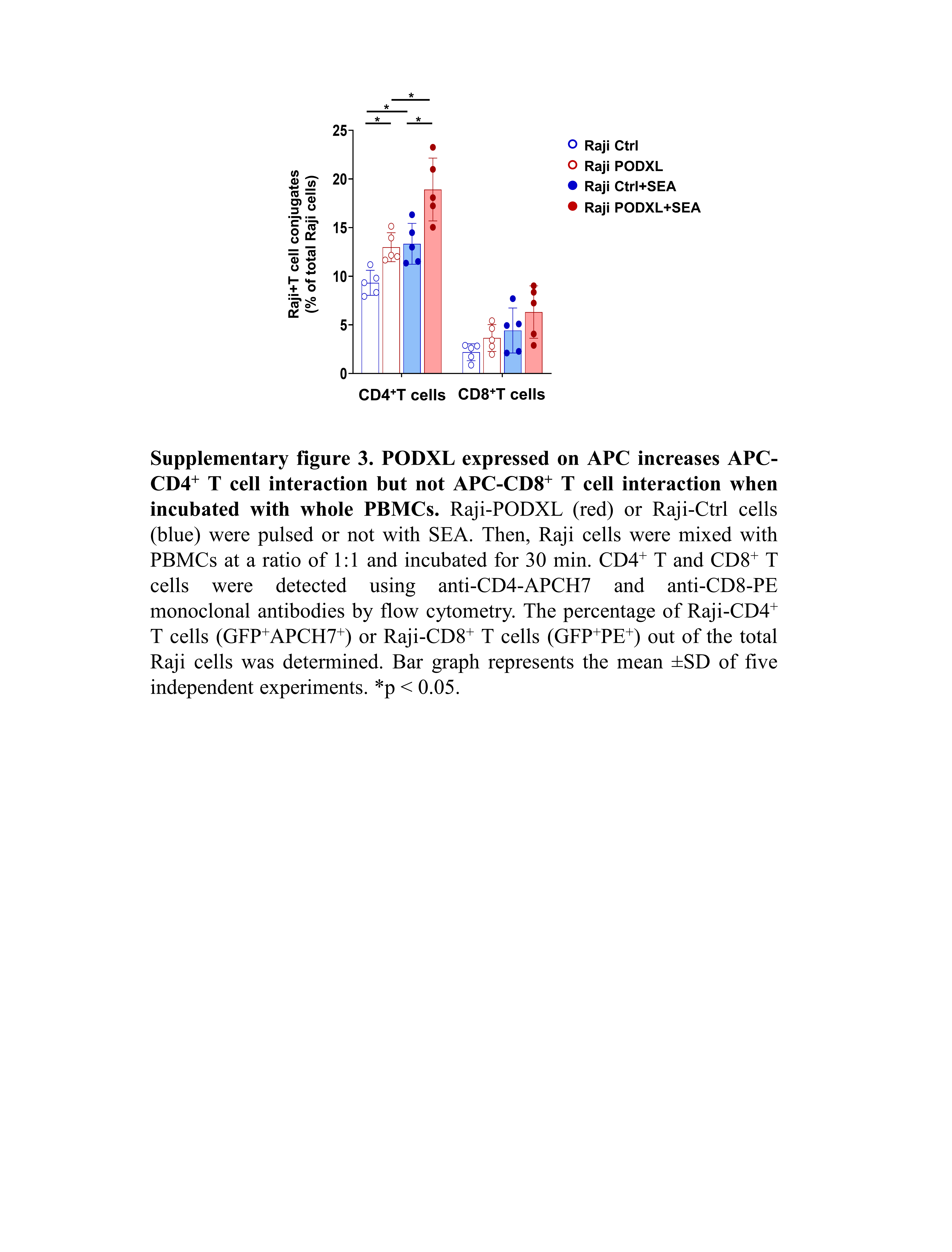

Supplement: Supplementary file 3 [file Image_3.tif]
